# Advancing the Management of Skull Base Chondrosarcomas: A Systematic Review of Targeted Therapies

**DOI:** 10.3390/jpm14030261

**Published:** 2024-02-28

**Authors:** Edoardo Agosti, Marco Zeppieri, Sara Antonietti, Tamara Ius, Marco Maria Fontanella, Pier Paolo Panciani

**Affiliations:** 1 Division of Neurosurgery, Department of Medical and Surgical Specialties, Radiological Sciences and Public Health, University of Brescia, Piazza Spedali Civili 1, 25123 Brescia, Italy; edoardo_agosti@libero.it (E.A.);; 2 Department of Ophthalmology, University Hospital of Udine, p.le S. Maria della Misericordia 15, 33100 Udine, Italy; 3 Neurosurgery Unit, Head-Neck and NeuroScience Department, University Hospital of Udine, p.le S. Maria della Misericordia 15, 33100 Udine, Italy

**Keywords:** chondrosarcomas, target therapies, molecular patterns, systematic reviews, outcomes

## Abstract

Background: Chondrosarcomas rank as the second most common primary bone malignancy. Characterized by the production of a cartilaginous matrix, these tumors typically exhibit resistance to both radiotherapy (RT) and chemotherapy (CT), resulting in overall poor outcomes: a high rate of mortality, especially among children and adolescents. Due to the considerable resistance to current conventional therapies such as surgery, CT, and RT, there is an urgent need to identify factors contributing to resistance and discover new strategies for optimal treatment. Over the past decade, researchers have delved into the dysregulation of genes associated with tumor development and therapy resistance to identify potential therapeutic targets for overcoming resistance. Recent studies have suggested several promising biomarkers and therapeutic targets for chondrosarcoma, including isocitrate dehydrogenase (IDH1/2) and COL2A1. Molecule-targeting agents and immunotherapies have demonstrated favorable antitumor activity in clinical studies involving patients with advanced chondrosarcomas. In this systematic review, we delineate the clinical features of chondrosarcoma and provide a summary of gene dysregulation and mutation associated with tumor development, as well as targeted therapies as a promising molecular approach. Finally, we analyze the probable role of the tumor microenvironment in chondrosarcoma drug resistance. Methods: A systematic search was conducted across major medical databases (PubMed, Embase, and Cochrane Library) up to 10 November 2023. The search strategy utilized relevant Medical Subject Heading (MeSH) terms and keywords related to “chondrosarcomas”, “target therapies”, “immunotherapies”, and “outcomes”. The studies included in this review consist of randomized controlled trials, non-randomized controlled trials, and cohort studies reporting on the use of target therapies for the treatment of chondrosarcoma in human subjects. Results: Of the initial 279 articles identified, 40 articles were included in the article. The exclusion of 140 articles was due to reasons such as irrelevance, non-reporting of selected results, systematic literature review or meta-analysis, and lack of details on the method/results. Three tables highlighted clinical studies, preclinical studies, and ongoing clinical trials, encompassing 13, 7, and 20 studies, respectively. For the clinical study, a range of molecular targets, such as death receptors 4/5 (DR4 and DR5) (15%), platelet-derived growth factor receptor-alpha or -beta (PDGFR-α, PDGFR-β) (31%), were investigated. Adverse events were mainly constitutional symptoms emphasizing that to improve therapy tolerance, careful observation and tailored management are essential. Preclinical studies analyzed various molecular targets such as DR4/5 (28.6%) and COX-2 (28.6%). The prevalent indicator of antitumoral activity was the apoptotic rate of both a single agent (tumor necrosis factor-related apoptosis-inducing ligand: TRAIL) and double agents (TRAIL-DOX, TRAIL-MG132). Ongoing clinical trials, the majority in Phase II (53.9%), highlighted possible therapeutic strategies such as IDH1 inhibitors and PD-1/PD-L1 inhibitors (30.8%). Conclusions: The present review offers a comprehensive analysis of targeted therapeutics for skull base chondrosarcomas, highlighting a complex landscape characterized by a range of treatment approaches and new opportunities for tailored interventions. The combination of results from molecular research and clinical trials emphasizes the necessity for specialized treatment strategies and the complexity of chondrosarcoma biology.

## 1. Introduction

Chondrosarcomas, arising from transformed cartilaginous cells, manifest in various skeletal sites, such as long bones, pelvis, and ribs, including the challenging subset of skull base chondrosarcomas [1]. Despite diagnostic advancements and an improved understanding of molecular underpinnings, optimal management remains elusive [2].

The rarity of chondrosarcomas, approximately 1 in 200,000 individuals, limits available data, with skull base chondrosarcomas representing a smaller fraction and posing diagnostic and therapeutic challenges [3]. Their location within the skull base, encroaching upon critical structures, amplifies the intricacy of clinical management [4].

Chondrosarcomas, including skull base cases, display resistance to traditional therapies, primarily managed through surgical resection. However, challenges arise due to proximity to vital structures and the risk of neurological deficits [5]. The relative insensitivity to conventional CT and RT further constrains treatment options, resulting in a notably poor prognosis, necessitating a shift in treatment approaches [4].

Recent studies focus on identifying molecular targets in chondrosarcomas, revealing intricate genetic aberrations, such as mutations in IDH genes, and dysregulation of signaling pathways like Hedgehog, mTOR, and vascular endothelial growth factor (VEGF) [6,7]. Despite incremental progress in unraveling molecular intricacies, translating this knowledge into effective therapeutic interventions remains challenging [8].

The pursuit of targeted therapies holds promise, offering a tailored approach addressing specific molecular aberrations and potentially overcoming resistance [8]. Considering the evolving landscape of targeted therapies, a systematic literature review becomes imperative. This review aims to critically evaluate existing studies on targeted therapies for chondrosarcomas, with specific attention to skull base cases, consolidating dispersed information and providing insights for future research endeavors.

## 2. Materials and Methods

### 2.1. Literature Review 

The PRISMA guidelines were adhered to during the execution of the systematic review [9]. Two investigators (E.A. and S.A.) meticulously conducted an exhaustive exploration of the literature using the databases PubMed, Ovid MEDLINE, and Scopus. The initial search transpired on 20 December 2023, with an update on 14 January 2024. A comprehensive search strategy was devised by combining various keywords, such as “chondrosarcomas”, “targeted therapies”, “outcomes”, and “adverse events”, utilizing both AND and OR combinations. Retrieval of studies employed MeSH terms and Boolean operators: (chondrosarcomas) AND (targeted therapies OR targeted treatments) AND (outcomes OR survival OR adverse events). Additional relevant articles were pinpointed through scrutinizing the references of selected papers. Inclusion criteria for study selection encompassed (1) English language; (2) in vitro, in vivo, or ex vivo investigations on targeted therapies for skull base chondrosarcomas; and (3) studies providing insights into clinical outcomes and/or adverse events. Conversely, exclusion criteria included (1) editorials, literature reviews, and meta-analyses and (2) studies lacking clear delineation of methods and/or results.

The inventory of identified studies was integrated into Endnote X9, where duplicate entries were expunged. Results were meticulously scrutinized independently by two researchers (E.A. and S.A.) adhering to the predefined inclusion and exclusion criteria. Any disparities were arbitrated by a third reviewer (P.P.P.). Subsequently, articles meeting the eligibility criteria underwent a thorough examination during the full-text screening process.

### 2.2. Data Extraction

Each study’s details were systematically extracted, encompassing the following information: authorship, publication year, patient cohort size, previous therapeutic interventions, targeted molecular entity, studied agent, supplementary interventions, clinical endpoints (encompassing progression-free survival (PFS), median PFS (mPFS), dimensions of lesions pre- and post-treatment), and reported adverse events. 

### 2.3. Outcomes

The primary outcomes focused on characterizing the main targeted treatments (including target, agent, dosage, and duration of treatment), accessible for skull base chondrosarcomas. Secondary outcomes encompassed clinical outcomes (i.e., disease control and progression-free survival—PFS) and the identification of adverse events associated with these interventions.

### 2.4. Risk of Bias Assessment

**Figure 1:**
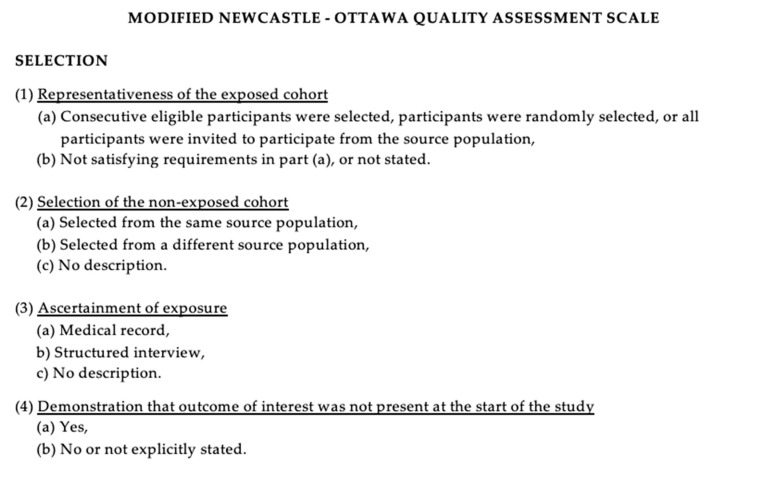

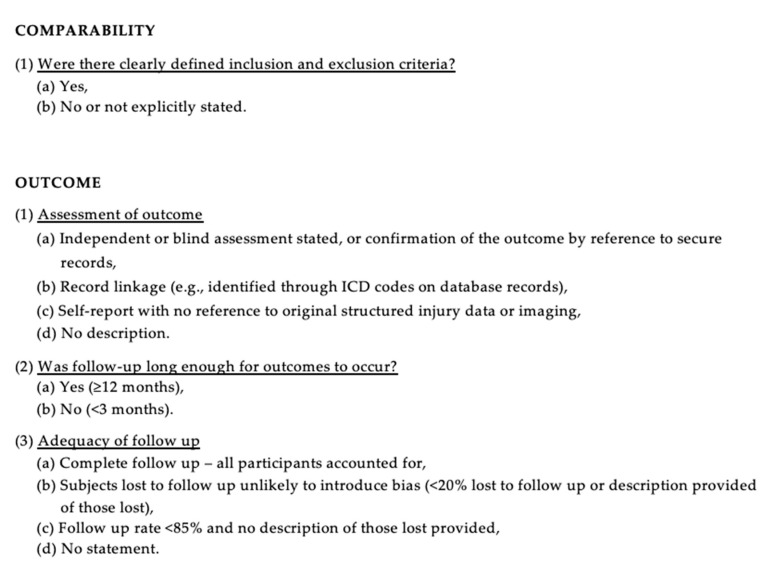
The modified NOS.

The evaluation of study quality was conducted using the Newcastle–Ottawa Scale (NOS) [10], which appraised the included studies based on selection criteria, comparability, and outcome assessment. Quality appraisal involves the assessment of the aforementioned aspects, with an optimal score being 9. Elevated scores denoted superior study quality, with studies garnering 7 or more points classified as high-quality. The quality assessment was independently conducted by two authors (E.A. and P.P.P.), and any disparities were resolved through re-examination by the third author (Figure 1).

### 2.5. Statistical Analysis

Ranges and percentages were included in the descriptive statistics that were provided. The R statistical software, version 3.4.1, was used for all statistical studies (http://www.r-project.org (accessed on 16 January 2024).

## 3. Results

### 3.1. Literature Review 

**Figure 2:**
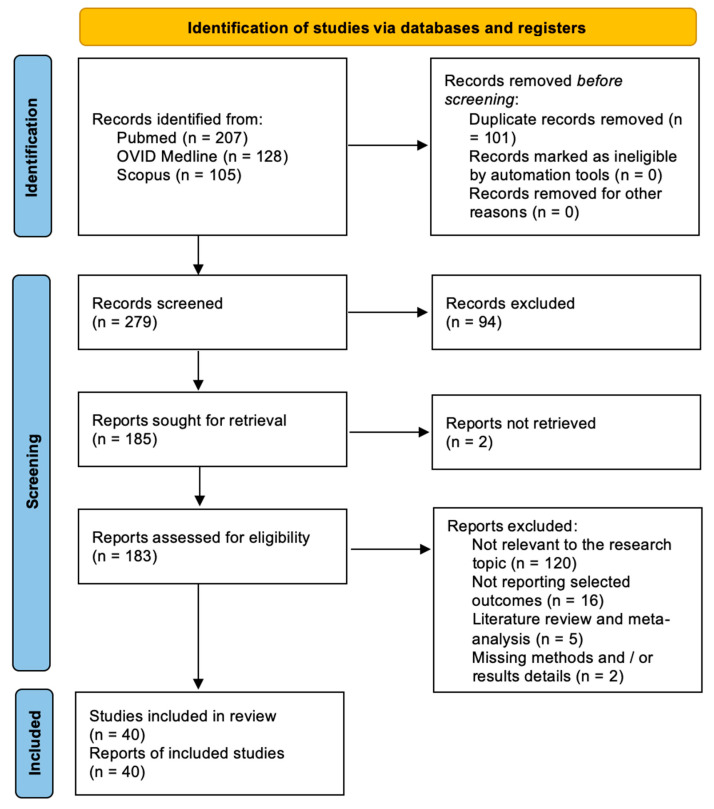
PRISMA flow chart.

After duplicates were eliminated, 279 papers in total were found. A number of 185 articles were found for full-text analysis after title and abstract analysis. For 183 articles, eligibility was determined, and for 40 articles, it was evaluated. The following criteria led to the exclusion of the remaining 140 articles: There are 120 publications that are not related to the study issue, 16 papers that do not provide selected outcomes, 5 articles that do not provide a systematic literature review or meta-analysis, and 2 articles that do not provide methodological or result information. For each of the patient groups under consideration, at least one or more outcome measures were available for all of the studies that were part of the analysis. The PRISMA statement’s flow chart is shown in Figure 2.

**Figure A1:**
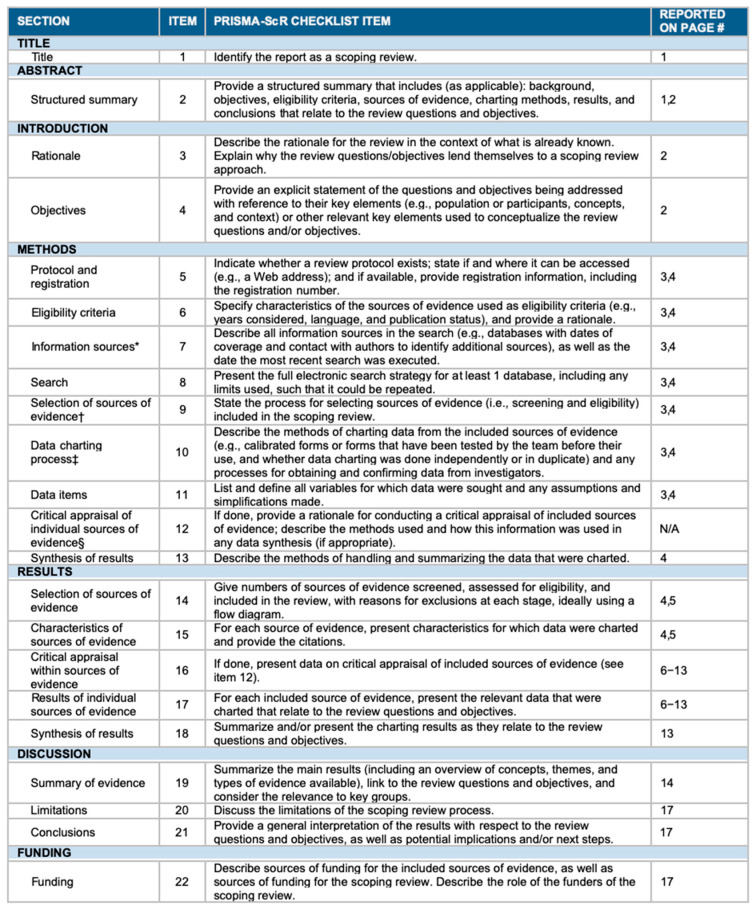
The PRISMA-ScR checklist. Abbreviations: JBI = Joanna Briggs Institute; PRISMA-ScR = Preferred Reporting Items for Systematic reviews and Meta-Analyses extension for Scoping Reviews. * Where sources of evidence (see second footnote) are compiled from, such as bibliographic databases, social media platforms, and Web sites. † A more inclusive/heterogeneous term used to account for the different types of evidence or data sources (e.g., quantitative and/or qualitative research, expert opinion, and policy documents) that may be eligible in a scoping review as opposed to only studies. This is not to be confused with information sources (see first footnote). ‡ The frameworks by Arksey and O’Malley and Levac and the JBI guidance refer to the process of data extraction in a scoping review as data charting. § The process of systematically examining research evidence to assess its validity, results, and relevance before using it to inform a decision. This term is used for items 12 and 19 instead of “risk of bias” (which is more applicable to systematic reviews of interventions) to include and acknowledge the various sources of evidence that may be used in a scoping review (e.g., quantitative and/or qualitative research, expert opinion, and policy document).

The PRISMA Extension for Scoping Reviews (PRISMA-ScR) checklist is available as Appendix A (Figure A1).

### 3.2. Data Analysis

A summary of the included studies reporting on targeted therapies for skull base chondrosarcomas is presented in Table 1, Table 2 and Table 3 for clinical study, preclinical studies, and ongoing clinical trials, respectively.

#### 3.2.1. Clinical Studies

A total number of 13 studies have been included. The studies encompassed in the systematic review span from 2010 to 2021, representing a decade-long exploration of targeted therapies for skull base chondrosarcomas. The patient cohort sizes displayed considerable variability across studies. Notably, single-patient studies were observed in 7.7% of cases, underscoring the rarity of skull base chondrosarcomas. Larger cohorts, such as the one involving 47 patients in a specific study, indicated concerted efforts to accumulate more extensive data, although this pattern was not consistently prevalent. Surgical interventions emerged as the most frequently reported prior treatments, occurring in 92.3% of cases. CT and RT were also prevalent but exhibited variations across studies.

The agents employed for targeted therapy included rhApo2L/TRAIL, Imatinib, Dulanermin, Cixutumumab/Temsirolimus, GDC-0449, Nivolumab, Dasatinib, Pembrolizumab, Pazopanib, Apatinib, Ivosidenib, and Regorafenib/Placebo. 

Clinical outcomes were evaluated in terms of PFS, mPFS, progressive disease (PD), and stable disease (SD). The mPFS exhibited a range from 5.5 months to 19.9 months [11,12], illustrating substantial variability in treatment responses. Adverse events associated with targeted therapies were consistently reported across studies, predominantly involving systemic manifestations. Common adverse effects included fatigue, anorexia, thrombocytopenia, diarrhea, hypertension, and nausea. The frequencies of these effects exhibited variations, without a specific prevalence of any particular adverse effect (Table 1).

**Table 1:** Summary of clinical studies included in the systematic literature review reporting on skull base chondrosarcomas.

Author, Year	Patients (N)	Age (Mean–Range)	Sex (F: M Ration)	Prior Treatment	Systemic Targeted Treatment				Next Treatment	Outcome	Adverse Effect
					Target	Agent	Dosage	Duration (Months)			
Herbst et al. [13] 2010	2	56 (53–59)	1:1	Surgery, CT (Irinotecan, Gemcitabine/Docetaxel, and Thalidomide), RT	DR4 and DR5	rhApo2L/TRAIL	8 mg/kg and 30 mg/kg	N/A	N/A	PR (3 mo and 10 mo after starting target therapy)	N/A
Grignani et al. [14] 2010	26	52 (23–81)	9:17	CT (Doxorubicine 20 pts, Cisplatin/Ifosfamide 6 pts)	PDGFR-α and PDGFR-β	Imatinib	400 mg BID	24	N/A	PD (18 pts) SD (8 pts)	N/A
Subbahia et al. [15] 2012	1	65	0:1	Surgery, RT, CT (6 cycles of Irinotecan)	DR4 and DR5	Dulanermin	8 mg/kg IV on days 1 through 5 in a 21-day cycle	N/A	Surgery, Dulanermin (16 mo)	NED	N/A
Schwartz et al. [16] 2013	17	45.5 (18–73)	10:7	Surgery	IGFIR, TOR	Cixutumumab/Temsirolimus	6 mg/kg IV/25 mg IV	12	N/A	mPFS: 6 mo; 3 mo PFS 31% in IGFR+ pt/ 39% in IGFR—pt,	Anemia (16 pts), hyperglycemia (18 pt), hypophosphatemia (16 pts), lymphopenia (25 pts), oral mucositis (19 pts), thrombocytopenia (19 pts)
Italiano et al. [17] 2013	45	56 (27–85)	14:31	N/A	Hh signal pathway	GDC-0449	150 mg per os, QD, in a 28-day cycle.	6	N/A	SD ≥ 6 mo (10 pts) PD (29 pts)	Dysgeusia (29 pts), fatigue (22 pts), myalgia (22 pts), alopecia (18 pts), ALT or AST increase (2 pts).
Paoluzzi et al. [18] 2016	1	74	N/A	N/A	PD-1	Nivolumab	3 mg/kg IV every 2 weeks	12	N/A	PR	N/A
Schuetze et al. [11] 2016	11	54 (22–87)	6:5	N/A	c-KIT, BCR-ABL, PDGFR-α and PDGFR-β	Dasatinib	70–100 mg BID per os.	1–87	N/A	mPFS: 5.5 mo, 6-mo PFS: 47%,	Fatigue, fever, anorexia, weight loss, rash, nausea, vomiting, constipation (1 pt)
Tawbi et al. [19] 2017	5	35 (22–48)	2:3	N/A	PD-L1	Pembrolizumab	200 mg	12	N/A	PR (1 pt), SD (1 pt), PD (3 pts)	Anemia (1 pt), decreased lymphocyte count (1 pt), prolonged APTT (1 pt), decreased platelet count (1pts)
Bupathi et al. [20] 2017	2	N/A	N/A	ST (Sunitinib, Nivolumab, Everolimus), RT	VEGFR-1/2/3, PDGFR, cKIT	Pazopanib	800 mg per os QD	15–19	Pazopanib (400 mg/day → 800 mg/day) QD and Temozolomide (150 mg/m^2^, 7 days on with 7 days off) in a 28-day cycle	PD, SD	Fatigue (1 pt), anorexia (1 pt), constipation (1 pt), hypertension (2 pts), thrombocytopenia (1 pt)
Chow et al. [21] 2020	47	58 (32–87)	18:29	Surgery, CT, RT	VEGFR-1/2/3, PDGRF, cKIT	Pazopanib	800 mg per os QD in a 28-day cycle	48	N/A	PR (1 pt), SD (30 pts), PD (11 pts) mPFS:7,9 mo, 3-mo PFS: 40%	ARF (1 pt), ALT elevation (4 pts), anemia (1 pt) diarrhea (1 pt) dyspnea (1 pt), fatigue (1 pt), hemorrhage CNSa (1 pt), hyperbilirubinemia (1 pt), hypertension (12 pts), hyponatremia (1 pt), left pulmonary vein thrombosis (1 pt), proteinuria(1 pt), pulmonary emboli (2 pt), thromboembolic event (1 pt)
Xie et al. [22] 2020	33	44,5 (17–72)	9:24	Surgery, CT (Doxorubicin, Ifosfamide in 13 pts)	VEGFR-2	Apatinib	500 mg per os QD, 30 min after the meal	N/A	N/A	PR (6 pts), SD (23 pts), PD (4 pts), mPFS of 7 mo, 6-mo PFS: 47%,	Anorexia (12 pts), wound dehiscence and infections (9 pts), platelet decrease (3 pts) hypertension (2 pts)
Tap W.D. et al. [23] 2020	21	55 (30–88)	8:13	Surgery (16 pts), ST (11 pts), RT (7 pts)	mutant IDH1	Ivosidenib	100 mg BID and 300–1200 mg QD per os in 28-day cycles	>47	N/A	SD 11 pts PD 6 pts mPFS: 5,6 mo 6 mo PFS: 39.5%	Diarrhea (9 pts), nausea (7 pts), fatigue (6 pts), edema peripheral (6 pts), upper respiratory tract infection (5 pts), constipation (4 pts), decreased appetite (4 pts), pain in extremity (5 pts), anemia (4 pts), arthralgia (3 pts), headache (3 pts), dizziness (3 pts), dyspnea (3 pts), vomiting (3 pts).
Duffaud et al. [12] 2021	40	64 (37.5–67.5)	15:25	CT (Doxorubicine, Ifosfamide, Cisplatin)	VEGFR1-3, TIE2, PDGFRβ, FGF, KIT, RET, RAF	Regorafenib/ Placebo	160 mg per os	53	N/A	PR 2 pts SD 16 pts PD 21 pts mPFS: 19,9/8 mo 6 mo PFS: 43/25%	Pain (31 pts), hypertension (13 pts), asthenia (24 pts), thrombocytopenia (5 pts), diarrhea (18 pts)

Abbreviations: APTT = prolonged activated partial thromboplastin time; ARF = acute renal failure; BID = twice a day; CNS = central nervous system; CT = chemotherapy; DR4/5 = death receptor 4/5; Hh = Hedgehog; IV = intravenous; mPFS = median PFS; mo = months; N/A = not applicable; NED = no evidence of disease; PD = progressive disease; PDGFR-α/β = Platelet-derived growth factor receptor; PR = partial response; QD = once daily; PFS = progression-free survival; RT = radiotherapy; SD = stable disease; SIR = sirolimus, ST = systemic therapy; TOR = target of rapamycin; TRAIL = tumor necrosis factor-related apoptosis-inducing ligand; VEGFR = vascular endothelial growth factor receptor.

#### 3.2.2. Preclinical Studies

A total number of seven studies have been included. Authorship and the publication year are crucial indicators of the evolution of research in this domain. The studies included in the review span several years (2003–2022), showcasing a continuum of scientific exploration. Tomek et al. [24], Fong et al. [25], Schrage et al. [26,27] Cheong et al. [28], Miladi et al. [29], and Higuchi et al. [30] all employed in vitro methodologies to investigate targeted treatments against skull base chondrosarcomas. 

Agents employed in the studies were diverse, including TRAIL, 2-Methoxyestradiol (2-ME), Imatinib, Dasatinib, Celecoxib, quaternary ammonium doxorubicin (QA-Dox), and Zaltoprofen. 

Proapoptotic effects, antitumor mechanisms, molecular target profiling, and the impact of selective inhibitors on chondrosarcoma growth were among the diverse study purposes identified. Apoptotic rates were a common metric, with varying percentages observed in response to different treatments. For instance, TRAIL alone exhibited a 20% apoptotic rate, while the combination with doxorubicin (TRAIL-DOX) resulted in a remarkable increase to 90–95%. Dasatinib demonstrated a 50% apoptotic rate, and COX-2 inhibitors, such as Celecoxib, showed a decrease in proliferation of chondrosarcoma in vitro (Table 2).

**Table 2:** Summary of preclinical studies included in the systematic literature review reporting on skull base chondrosarcomas.

Author, Year	Study Type	Targeted Treatment			Study Purpose	Results
		Target	Agent	Dosage		
Tomek et al. [24] 2003	In vitro	DR4, DR5, TRID, TRUNDD, osteoprotegerin	TRAIL	100–1000 ng/mL	Proapoptotic effect of TRAIL alone or in combination with conventional CT	TRAIL: 20% apoptotic rate TRAIL-DOX: 90–95% apoptotic rate
Fong et al. [25] 2006	In vitro	HIF-1α	2-ME	0–20 μM	Mechanism of antitumor activity of 2 ME on human chondrosarcoma	Cells accumulated in the G0/G1 phase in response to 2 ME and DAPI stain indicated an induction of apoptosis
Schrage et al. [26] 2009	In vitro	PDGFR	Imatinib, Dasatinib	1.0–100 μmol/L, 5.0–1.0 μmol/L	Molecular targets for systemic treatment of chondrosarcoma using kinome profiling	Dasatinib: 50% apoptotic rate chondrosarcoma does not respond to imatinib treatment in vitro
Schrage et al. [27] 2009	In vitro	COX-2	Celecoxib	5–25 μM	Effect of selective COX-2 inhibition on chondrosarcoma growth	COX-2 inhibitors decrease the proliferation of chondrosarcoma in vitro
Cheong et al. [28] 2011	In vitro	DR4, DR5, TRID, TRUNDD, osteoprotegerin	TRAIL	10–20 ng/mL	Proapoptotic effect of TRAIL alone or in combination with proteosome inhibitor MG132	TRAIL: 20% apoptotic rate TRAIL-MG132: 60% apoptotic rate
Miladi et al. [29] 2017	In vitro	MMP	QA-Dox	25–300 µM	MMP inhibitors were conjugated with a QA function as a targeting ligand to proteoglycans of the chondrosarcoma extracellular matrix	In the chondrosarcoma model, the MMP13 inhibitor Dox and its QA derivative are promising as adjuvant therapies for chondrosarcoma management
Higuchi et al. [30] 2022	In vitro	COX-1 and COX-2	Zaltoprofen	0–400 µmol/L	Expression of PPARγ at the mRNA and protein levels, following the induction of PPARγ-activating factors	Inhibition of proliferation of H-EMC-S5 cells observed in vitro

Abbreviations: COX1-2 = cyclooxygenases 1–2; CT = chemotherapy; DAPI = 4′,6-diamidino-2-phenylindole; DOX = doxorubicin; DR4/DR5 = death receptors 4/5; H-EMC-S5 = human extraskeletal chondrosarcoma; 2-ME = 2-methoxyestradiol; MMP = matrix metalloproteinase; N/A = not applicable; PDGFR = platelet-derived growth factor receptors; PPARγ = peroxisome proliferator-activated receptors; QA-Dox = quaternary ammonium doxorubicin; TRAIL = tumor necrosis factor-related apoptosis-inducing ligand.

**Table 3:** Summary of ongoing clinical trials included in the systematic literature review reporting on skull base chondrosarcomas.

NCT Number	Year	Phase	Agent Classes	Agents	Target
NCT01267955	2010	II	Hh pathway inhibitor	Vismodegib	Smo
NCT01883518	2013	I–II	Cell therapy	Autologous dendritic cell vaccine	TA
NCT02821507	2014	II	mTOR inhibitor, CT	Sirolimus, Cyclophosphamide	mTOR
NCT03277924	2017	I–II	Antiangiogenic, PD-L1 inhibitor, CT	Sunitinib, Nivolumab, Epirubicin, Ifosfamide, Doxorubicin, Dacarbazine, Cisplatin, Methotrexate	RTKs PD-1
NCT02982486	2017	II	CTLA-4 inhibitor, PD-L1 inhibitor	Ipilimumab Nivolumab	CTLA-4, PD-L1
NCT03474640	2018	I	PD-1 inhibitor	Toripalimab	PD-1
NCT03449108	2018	II	Recombinant IL-2, cell therapy, CT, CTLA-4 inhibitor, PD-L1 inhibitor	Aldesleukin, Autologous tumor-infiltrating lymphocytes LN-145, Autologous tumor-infiltrating lymphocytes LN-145-S1, Cyclophosphamide, Fludarabine, Ipilimumab, Nivolumab	IL-2Rβ CTLA-4 PD-L1
NCT03715933	2018	I	Antibody targeting DR5, CT	INBRX-109 Carboplatin Cisplatin Pemetrexed 5-fluorouracil Irinotecan Temozolomide	DR5
NCT03684811	2018	I–II	IDH1 inhibitor	FT-2102 + azacitidine	IDH1
NCT03670069	2019	I	JAK-1 inhibitor	Itacitinib	JAK-1
NCT04040205	2019	II	CDK4/6 inhibitor	Abemaciclib	CDK4/6
NCT04278781	2020	II	IDH1 inhibitor	AG-120	IDH1
NCT04340843	2020	II	HDAC inhibitor, antimetabolites	Belinostat, Decitabine, Cedazuridine, Guadecitabine	HDAC
NCT04553692	2020	I	Antibody targeting DR5, CT SMAC, inhibitor of IAP, BCL2 inhibitor	IGM-8444 (Aplitibart) FOLFIRI Bevacizumab (and approved biosimilars) Birinapant Venetoclax Gemcitabine Docetaxel Azacitidine	DR5 IAP BCL2
NCT04690725	2020	I–II	PI3Ka inhibitor	TQB3525	PI3Ka
NCT04521686	2020	I	IDH1 and IDH2 inhibitor	LY3410738	IDH1 and IDH2
NCT05131386	2021	II	CT	Trabectedin	DNA
NCT04762602	2021	I	IDH1 and IDH2 inhibitor	HMPL-306	IDH1 and IDH2
NCT05039801	2021	I	Glutaminase-1 inhibitor, PD-1 inhibitor	IPN60090 Bevacizumab Paclitaxel Capivasertib	Glutaminase-1 PD-1
NCT04950075	2021	II	Tetravalent DR5 agonistic antibody	INBRX-109	DR5

Abbreviations: BCL2 = B-cell leukemia/lymphoma 2 protein; CT = chemotherapy; CTLA-4 = cytotoxic T-lymphocyte antigen 4; DR4/5 = death receptors 4/5; HDAC = histone deacetylase inhibitors; Hh = Hedgehog; IAP = inhibitor of apoptosis protein; IDH1/2 = isocitrate dehydrogenase 1/2; IL-2Rβ = interleukin 2 receptor β chain; mTOR = mammalian target of rapamycin; PD-1 = programmed cell death protein 1; PD-L1 = programmed cell death ligand 1; PI3K = phosphoinositide 3-kinases; SMAC = second mitochondrial-derived activator of caspases; RTK = receptor tyrosine kinases; SMO = smoothened protein; TA = tumor antigen.

#### 3.2.3. Ongoing Clinical Trials

A total number of 20 studies have been included. The publication years of the ongoing clinical trials span a range, indicating a continuous and evolving effort over time, with studies published from 2010 to 2021. The majority of trials were in Phase II, representing 53.85% of the total trials, followed by Phase I, accounting for 38.46%, and Phase I–II at 23.08%. This distribution suggests a significant emphasis on evaluating the efficacy and safety of targeted therapies in a broader patient population, signaling a crucial stage in the developmental trajectory of these interventions.

Turning to agent classes, IDH1 inhibitors and PD-1/PD-L1 inhibitors emerged as the most prevalent, each constituting 30.8% of the trials. These classes are closely followed by CTLA-4 inhibitors, histone deacetylase inhibitors (HDAC) inhibitors, and cell therapy, each at 15.4%. This diversity underscores the multifaceted nature of targeted therapies under investigation, reflecting a comprehensive approach to addressing the complexities of skull base chondrosarcomas. Examining specific agents, INBRX-109 and Nivolumab are the most frequently studied, each featuring in 23.08% of the trials. Ipilimumab, Vismodegib, and autologous dendritic cell vaccine are each represented in 15.4% of the trials. Notably, these specific agents span various agent classes, highlighting the cross-disciplinary nature of the therapeutic strategies being explored.

## 4. Discussion

Chondrosarcoma, a rare malignant tumor of cartilaginous origin, poses a considerable challenge in terms of treatment due to its resistance to conventional therapies. In recent years, efforts have been directed toward identifying targeted therapies that may offer improved outcomes for patients. This systematic literature review shed light on several potential avenues for the treatment of chondrosarcomas. In detail, IDH1 inhibitors, growth factor receptor inhibitors, and PD-1/PD-L1 inhibitors emerged as the most promising and studied, followed by CTLA-4 inhibitors, histone deacetylase inhibitors, and cell therapy.

### 4.1. Targeted Therapies for Skull Base Chondrosarcomas

#### 4.1.1. Trabectedin and Genomic Landscapes

In the pursuit of effective targeted therapies for chondrosarcomas, the exploration of trabectedin presents a particularly promising avenue. Morioka et al. [31] conducted a phase 2 study that yielded encouraging results, specifically in the context of extraskeletal myxoid chondrosarcomas and mesenchymal chondrosarcomas. These subtypes, often challenging to treat, exhibited positive responses to trabectedin, suggesting its potential as a therapeutic option for these specific patient populations [31].

Moreover, the study by Nacev et al. [32] has significantly contributed to our understanding of the genomic landscapes in soft tissue and bone sarcomas. Through clinical sequencing, the research unveiled a myriad of genetic variations, emphasizing the intricate heterogeneity that exists within chondrosarcomas. The identification of these diverse genomic landscapes is not only paramount for comprehending the underlying molecular mechanisms of the disease but also lays the foundation for developing precision medicine approaches [32]. The concept of tailoring therapies to individual patients gains significance in light of these findings.

Trabectedin, in this context, emerges not merely as a treatment option but as a prototype for the direction that personalized medicine can take in chondrosarcoma [33]. The positive outcomes observed in specific subtypes highlight the importance of identifying biomarkers that can predict treatment response. Integrating genomic information into clinical decision-making processes can aid in patient stratification, ensuring that individuals most likely to benefit from trabectedin and similar therapies receive them [33].

#### 4.1.2. Angiogenesis and Anti-Angiogenic Therapies

The intricate vascular dynamics within cartilage tumors, notably pathologic neovascularization, as highlighted by McGough et al. [34] underscore the potential role of angiogenesis in chondrosarcoma progression. This pathologic neovascularization contributes to the sustenance of the tumor microenvironment, supporting the aggressive growth observed in chondrosarcomas. Additionally, Ayala et al. [35] shed light on the microvasculature and VEGFR expression in cartilaginous tumors, reinforcing the significance of angiogenic processes in the tumor’s biology.

The recognition of angiogenesis as a key player in chondrosarcoma pathogenesis has prompted investigations into anti-angiogenic therapies as potential interventions. Among these, pazopanib, a multi-tyrosine kinase inhibitor, has been explored, with van der Graaf et al. [36] presenting findings from a randomized phase 3 trial (PALETTE) that demonstrated its efficacy in metastatic soft-tissue sarcoma. The success of pazopanib in targeting angiogenic pathways has opened avenues for its evaluation in chondrosarcoma, providing a rationale for considering anti-angiogenic agents as a viable therapeutic strategy [36].

In a retrospective multiple-institution study, Li investigated the efficacy and safety of anlotinib [37], another anti-angiogenic agent, in patients with unresectable or metastatic bone sarcoma. 

The consideration of anti-angiogenic therapies introduces a novel dimension to chondrosarcoma treatment, focusing not only on inhibiting tumor cell proliferation but also on disrupting the supportive microenvironment essential for tumor sustenance [38]. The success of pazopanib and anlotinib in other sarcomas prompts careful evaluation and dedicated clinical trials to ascertain their efficacy and safety specifically in chondrosarcoma, given the shared challenges posed by these malignancies. Moreover, the identification of specific biomarkers associated with angiogenesis in chondrosarcoma can aid in patient selection, ensuring that individuals with a higher likelihood of response benefit from these targeted interventions [21].

As the exploration of anti-angiogenic therapies progresses, it becomes imperative to consider their integration into multimodal treatment approaches. Combining anti-angiogenic agents with existing modalities, such as surgery or RT, holds the potential to enhance treatment outcomes by addressing multiple facets of chondrosarcoma biology [14,22,38].

#### 4.1.3. Growth Factor Receptors: Therapeutic Target

The pursuit of precision therapies in chondrosarcoma has led to significant strides in understanding and targeting specific growth factor receptors, as exemplified by the work of Grignani et al. [14] and Duffaud et al. [12].

Grignani et al. [14] conducted a phase 2 trial investigating the efficacy of imatinib mesylate in patients with recurrent nonresectable chondrosarcomas expressing PDGFR-α or -β. This study showcased the potential of targeting specific growth factor receptors in chondrosarcoma, providing evidence for the feasibility of tailored therapies based on the molecular characteristics of the tumor [14]. Imatinib mesylate, a tyrosine kinase inhibitor, demonstrated activity against PDGFRs, underlining the importance of identifying and selectively targeting receptors implicated in chondrosarcoma progression [39,40]. 

Moreover, regorafenib, another multi-kinase inhibitor, demonstrated efficacy in metastatic or locally advanced chondrosarcoma, as reported by Duffaud et al. [12] The success of regorafenib in a multicenter phase II study reinforces the relevance of growth factor receptor inhibition as a therapeutic strategy. Regorafenib’s ability to target multiple kinases, including those involved in angiogenesis and oncogenesis, aligns with the complex molecular landscape of chondrosarcoma [41,42].

The identification of specific receptors, such as PDGFRs, as potential therapeutic targets supports the use of existing drugs like imatinib mesylate and informs the development of novel agents with enhanced receptor specificity, aligning with the trend in oncology towards personalized and targeted therapies [43]. The success of imatinib mesylate and regorafenib in targeting growth factor receptors in chondrosarcoma opens avenues for further exploration, with clinical trials assessing their efficacy in combination with other modalities or in specific patient subpopulations providing additional insights [44]. Identifying biomarkers predictive of response to growth factor receptor inhibitors can refine patient selection, addressing challenges such as the heterogeneity of chondrosarcoma subtypes and the need for a comprehensive understanding of the interplay between different signaling pathways [3,5,7,9,10].

#### 4.1.4. Immunotherapy Approaches

In exploring immunotherapeutic strategies for chondrosarcoma, Chow et al. [21] investigated pazopanib’s efficacy in patients with surgically unresectable or metastatic chondrosarcoma. The study highlighted the potential of immunomodulation as a valuable component in the treatment landscape. Furthermore, the study conducted by Tawbi et al. [19] on pembrolizumab, showcasing its activity in advanced soft-tissue sarcoma and bone sarcoma, suggests a promising role for immunotherapy in the context of chondrosarcoma. These findings underscore the significance of incorporating immunotherapeutic approaches into the comprehensive management of chondrosarcoma, paving the way for further exploration of their effectiveness in clinical settings [18,45,46,47,48].

#### 4.1.5. IDH Mutations and Related Pathways Alteration

Understanding the molecular landscape of chondrosarcoma is crucial for identifying potential therapeutic targets, and recent research has provided valuable insights. Amary et al. [49] and Schaap et al. [50] have highlighted the frequency of IDH1 and IDH2 mutations in central chondrosarcomas, emphasizing the need to explore targeted interventions [51]. IDH mutations are not exclusive to chondrosarcoma; they are known to play pivotal roles in various cancers [25,52,53]. For instance, the study by Amary et al. [49] underscores the widespread relevance of IDH1 mutations in different cancer types.

Yang et al. investigated the expression of PD-L1/PD-L2 in chondrosarcoma, revealing an association with a high proliferation index of Ki-67, suggesting a potential link between immune checkpoint expression and cellular proliferation [54]. Iseulys et al. further elucidated the immune landscape, identifying an immunosuppressive environment in dedifferentiated subtypes and highlighting CSFR1+ macrophages as a promising therapeutic target [55]. These findings underscore the intricate interplay between the tumor microenvironment and immune responses in chondrosarcoma, laying the foundation for exploring immunotherapeutic strategies [56].

The genetic landscape of chondrosarcoma extends beyond IDH alterations. Tarpey et al. reported frequent mutations in the major cartilage collagen gene COL2A1, showcasing genetic diversity within the malignancy and presenting challenges and opportunities for targeted therapies [57]. Zhang et al. conducted functional profiling of receptor tyrosine kinases and downstream signaling, identifying potential pathways for rational targeted therapy [58].

Dysregulation of signaling pathways also plays a crucial role in chondrosarcoma progression. Gagné et al. explored the oncogenic activities of IDH1/2 mutations, emphasizing their impact on cellular signaling and highlighting the need for strategies targeting both the mutations and downstream signaling cascades [59].

#### 4.1.6. Epigenetic Vulnerabilities

Venneker et al.’s study highlights the crucial role of exploring epigenetic vulnerabilities in chondrosarcoma, going beyond the well-documented influence of IDH mutations [60]. Epigenetic dysregulation, a key aspect of cancer biology, contributes to the initiation and progression of various malignancies, making it paramount to understand these alterations in chondrosarcoma [61]. The study emphasizes the broader landscape of epigenetic vulnerabilities, detailing alterations in DNA methylation patterns, histone modifications, and chromatin remodeling processes impacting gene expression regulation [60]. Being dynamic and reversible, epigenetic modifications become attractive targets for therapeutic interventions, and the identification of specific regulators implicated in chondrosarcoma pathogenesis opens new possibilities for therapeutic strategies. Targeting regulators like DNA methyltransferases (DNMTs), HDACs, and chromatin remodeling enzymes could offer a unique approach to modulating gene expression patterns, potentially reversing or mitigating oncogenic processes in chondrosarcoma cells [60,62,63].

Recent advances in epigenetic-targeted therapies in other cancer types provide a promising framework for chondrosarcoma research. For instance, small molecule inhibitors targeting DNMTs or HDACs have shown efficacy in certain cancers by restoring normal epigenetic patterns and reactivating tumor-suppressor genes [64,65]. Applying similar strategies in chondrosarcoma may unveil novel avenues for therapeutic intervention. The intricate cross-talk between genetic mutations and epigenetic modifications highlights the need for a comprehensive approach that considers both aspects in the development of targeted therapies [66].

#### 4.1.7. Hippo-YAP/TAZ Signaling Pathway

The roles of YAP and TAZ in cancer, discussed by Moroishi et al., add molecular complexity to chondrosarcoma, known for their oncogenic influence in sarcomas [67]. Fullenkamp et al. highlight the frequent activation of YAP and TAZ oncoproteins in sarcomas, presenting them as potential therapeutic targets, particularly in chondrosarcoma where dysregulation of the Hippo-YAP/TAZ pathway contributes to uncontrolled cell growth [68]. Targeting this pathway emerges as a novel therapeutic approach, emphasizing the need for further research to unveil its full potential in chondrosarcoma [68]. Moya and Halder’s study elaborates on the Hippo-YAP/TAZ signaling axis in organ regeneration, suggesting a regenerative medicine perspective for treatment, linking the regulatory mechanisms of YAP and TAZ to both chondrosarcoma progression and innovative regenerative medicine approaches [69].

Recent advancements in cancer research have identified small molecules and biological agents capable of modulating the Hippo-YAP/TAZ pathway. These include inhibitors targeting YAP/TAZ transcriptional co-activators, upstream Hippo pathway components, or cross-talk molecules that influence pathway activity. The exploration of these inhibitors in preclinical models and early-phase clinical trials may pave the way for novel therapeutic strategies against chondrosarcoma [70,71]. It is crucial to assess the context-specific functions of YAP and TAZ in chondrosarcoma subtypes, considering potential heterogeneity in pathway activation among patients. 

### 4.2. Challenges, Considerations, and Future Developments

Despite the promising findings, challenges persist in translating these discoveries into effective clinical treatments. Heterogeneity within chondrosarcomas necessitates personalized approaches, considering the specific molecular alterations present in individual cases. The rarity of chondrosarcoma also poses challenges in conducting large-scale clinical trials [6].

Considerations for skull base chondrosarcoma, a subset with unique anatomical challenges, should be a focal point for future research. The proximity to critical structures in the skull base demands precision in treatment strategies to minimize collateral damage. Advanced imaging modalities and surgical techniques may play a crucial role in enhancing the management of skull base chondrosarcoma [4].

In accordance with our results, IDH1 inhibitors, growth factor receptor inhibitors, and PD-1/PD-L1 inhibitors seem to play pivotal roles in addressing the unique molecular characteristics of these tumors. IDH1 inhibitors, such as Ivosidenib and Vorasidenib, by disrupting the aberrant metabolic pathways associated with IDH1 mutations, hold promise in impeding tumor growth. Additionally, growth factor receptor inhibitors, including agents like imatinib and sunitinib, offer targeted intervention by disrupting signaling pathways crucial for chondrosarcoma development. Furthermore, the emergence of immune checkpoint inhibitors like pembrolizumab and nivolumab, which target the PD-1/PD-L1 axis, represents a significant breakthrough in unleashing the immune system against chondrosarcomas. These inhibitors hold the potential to overcome the immunosuppressive microenvironment of chondrosarcomas, fostering antitumor immune responses and improving patient outcomes in the realm of precision medicine for this challenging malignancy.

Future developments should prioritize collaborative efforts, pooling resources and data to better understand the molecular intricacies of chondrosarcoma. Innovative trial designs, incorporating novel endpoints and real-time molecular profiling, may expedite the evaluation of targeted therapies.

## 5. Conclusions

This systematic review of targeted therapies for skull base chondrosarcomas reveals a multifaceted landscape marked by diverse treatment modalities and emerging avenues for personalized interventions. The amalgamation of findings from clinical trials and molecular studies underscores the complexity of chondrosarcoma biology and highlights the need for tailored therapeutic approaches. While trabectedin exhibits promise in treating specific subtypes, the exploration of genomic landscapes by Nacev et al. [24] emphasizes the imperative of precision medicine in targeting individualized therapeutic vulnerabilities. Pathologic neovascularization, growth factor receptors, and immunotherapeutic strategies have emerged as crucial facets, fostering optimism in the development of effective interventions. Additionally, the identification of molecular alterations, such as IDH mutations, unveils potential targets, while the intricate interplay of epigenetic regulators and the activation of the Hippo-YAP/TAZ pathway present novel therapeutic avenues. Despite these advancements, challenges persist, including the heterogeneous nature of chondrosarcoma and the limited understanding of the optimal sequencing of therapies. As we navigate these challenges, ongoing research and future developments hold the promise of refining treatment strategies and enhancing outcomes for patients with skull base chondrosarcomas.

## Data Availability

Data are available in a publicly accessible repository.
